# Supplementary material for: Inhibition of Osteoclastogenesis by Thioredoxin-Interacting Protein-Derived Peptide (TN13)
Source: J Clin Med. 2019 Mar 29;8(4):431. doi: 10.3390/jcm8040431 (PMC6518213; doi:10.3390/jcm8040431)
Supplement: Supplementary file 1 [file jcm-08-00431-s001.pdf]

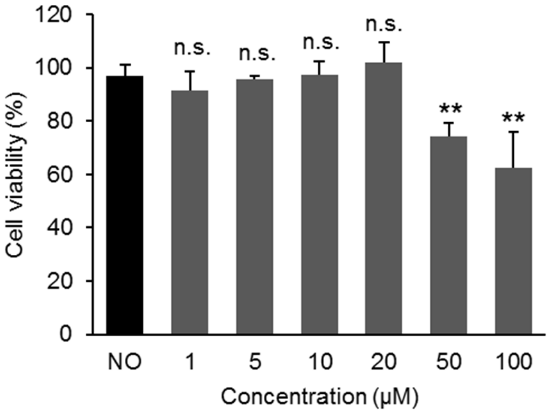

Figure S1. Viability of RAW 264.7 cells under various concentration of TAT-TN13. TAT-TN13 treated RAW 264.7 cells were tested by CCK8 assays. RAW 264.7 cells treated with the indicated concentrations of TAT-TN13 for 4 days (n=3). Repeated 3 times. The results are presented as the mean ± S.D of three independent experiments **P < 0.01; n.s.: not significant).

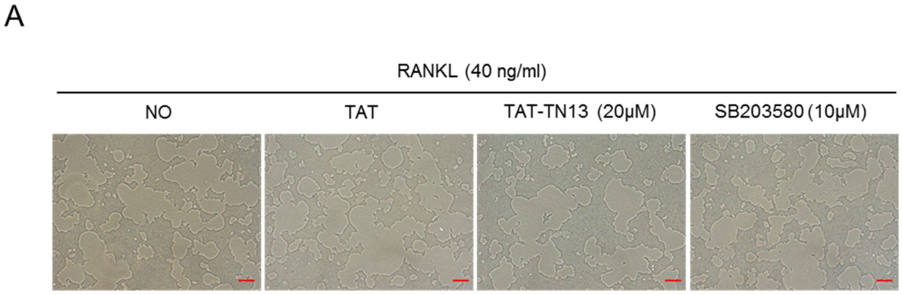

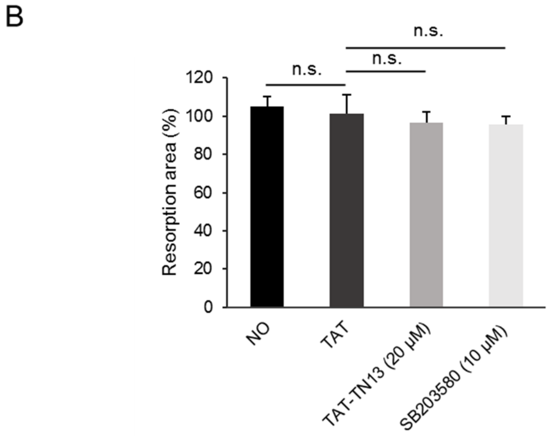

Figure S2. Bone resorbing activity of mature osteoclasts. RAW 264.7 cells were differentiated into osteoclasts by RANKL treatment for 4 days and we then treated TAT-TN13 (20 μM) or SB203580 (10 μM) for 12h. (A) Resorption pits on the plates were captured using a light microscope (IX71; Olympus) (scale bars: 50 μm). (B) Resorption pit areas were quantified using Olympus cellSens imaging software. The results are presented as the mean ± S.D of three independent experiments n.s.: not significant). NO: control, which was treated with PBS; TAT: treated control, which was treated with control peptide.
